# Combination Treatment of Docetaxel Liposomes and Sonodynamic Therapy With Verteporfin Liposomes in Patients With Cancer: A Report of Three Cases

**DOI:** 10.1155/crom/7515689

**Published:** 2026-08-30

**Authors:** Yasuo Komura, Shintarou Kimura, Yumi Hirasawa, Ryo Takasaki, Saki Fukuro, Nguyen Thi Mai Thi, Miyaji Gengou, Lee Sang Woo, Osamu Imataki, Toru Yanagawa, Koichiro Homma

**Affiliations:** ^1^ Rinku Medical Clinic, Osaka, Japan; ^2^ Department of Otolaryngology-Head and Neck and Oral-Maxillofacial Surgery, Institute of Medicine, Faculty of Medicine, University of Tsukuba, Tsukuba, Japan, tsukuba.ac.jp; ^3^ IGT Clinic, Osaka, Japan; ^4^ TCBIO Co. Ltd., Seoul, South Korea; ^5^ Central Clinical Laboratory, Kawasaki Medical School General Medical Center, Okayama, Japan, kawasaki-m.ac.jp; ^6^ Department of Emergency and Critical Care Medicine, Keio University School of Medicine, Tokyo, Japan, keio.ac.jp

**Keywords:** gastric cancer, liposome, lung cancer cells, ovarian metastases, sonodynamic therapy, verteporfin

## Abstract

Despite advances in medical technology, cancer treatment remains challenging. Our research focused on combining microtubule polymerization inhibitors with sonodynamic therapy (SDT) as a promising approach for cancer. SDT is a minimally invasive modality that uses tumor‐selective sonosensitizers to induce the death of malignant and tumor‐associated cells within the tumor microenvironment through targeted ultrasound irradiation. In this study, verteporfin was selected because of its dual properties of high ultrasound sensitivity and the ability to inhibit the yes‐associated protein. Liposome‐encapsulated docetaxel and verteporfin were employed to enhance tumor accumulation. This intervention demonstrated remarkable antitumor efficacy in three patients with ovarian metastases from gastric cancer, advanced gastric cancer, and lung metastases from breast cancer, respectively, without notable adverse events. Although the patients with ovarian and lung metastases had experienced progressive disease despite prior chemotherapy and immune checkpoint inhibitor treatment, our intervention successfully inhibited tumor progression. Furthermore, although conventional treatment options were considered unsuitable because of advanced age in the patient with gastric cancer, this intervention demonstrated a favorable safety profile and potential therapeutic benefit. To our knowledge, this study represents the first clinical report demonstrating the potential antitumor efficacy and safety of the combination of verteporfin liposomes, liposomal docetaxel, and SDT.

## 1. Introduction

Despite continuous advances in medical knowledge, the global incidence and mortality of cancer continue to rise [1]. Achieving a radical cure remains challenging because of multiple factors, including metastasis, drug resistance, and treatment discontinuation caused by adverse events [2]. To address these complex challenges associated with cancer treatment, we developed a therapeutic approach combining taxane‐based anticancer agents with sonodynamic therapy (SDT). SDT activates sonosensitizers, such as porphyrin compounds and phthalocyanine derivatives, through sonoluminescence generated by cavitation of nanobubbles. This process induces lipid peroxidation of cell membranes and oxidative damage to proteins through the generation of reactive oxygen species, including singlet oxygen, resulting in selective cytotoxicity in ultrasound‐targeted cancer cells [3–5]. Similar to photodynamic therapy (PDT), SDT is a noninvasive cancer treatment modality. However, ultrasound offers greater applicability for treating tumors across diverse anatomical sites owing to its superior tissue penetration compared with light [6]. This deeper penetration enables SDT to target tumors located at greater depths than PDT [7]. In addition, the use of focused ultrasound allows cavitation to be induced preferentially within the targeted tissue. As sonoluminescence is generated during cavitation events, activation of sonosensitizers can be spatially confined to the ultrasound focal zone [8, 9]. Notably, verteporfin—unlike other porphyrin‐based agents such as talaporfin sodium and porfimer sodium—inhibits the yes‐associated protein (YAP) and lexerts antiproliferative effects even in the absence of light irradiation [10–12].

Verteporfin has historically been used as a photosensitizer in PDT for the treatment of age‐related macular degeneration [13]. In recent years, its therapeutic potential has expanded beyond ophthalmology, attracting considerable interest as a sensitizer for both PDT and SDT in cancer therapy [12, 14]. Mechanistically, verteporfin inhibits the interaction between YAP and transcriptional enhanced associate domain (TEAD) transcription factors, thereby suppressing the formation of transcriptional complexes and the downstream expression of growth‐promoting genes [15]. Through YAP inhibition, verteporfin exerts intrinsic antitumor activity and may further enhance the therapeutic efficacy of both PDT and SDT [12]. Furthermore, accumulating preclinical evidence suggests that verteporfin‐mediated SDT may enhance antitumor immunity through multiple mechanisms, including immunogenic cell death (ICD) and modulation of the tumor immune microenvironment. Recent reviews have highlighted these immunomodulatory effects as important contributions to the therapeutic potential of SDT in solid tumors [16, 17]. In addition, verteporfin suppresses programmed death ligand 1 (PD‐L1) expression through inhibition of YAP, which may further strengthen antitumor immune responses [16].

Taxane‐based anticancer agents exert their antitumor effects by inhibiting the depolymerization of tubulin, a major component of microtubules, thereby stabilizing microtubules and inducing G2/M (mitotic) phase arrest [18, 19]. Reactive oxygen species generated during SDT can similarly induce G2/M phase arrest through DNA damage and oxidative stress [20]. Therefore, the combination of taxane‐based anticancer agents and SDT may simultaneously target cancer cells through distinct G2/M phase‐related mechanisms, including microtubule stabilization and oxidative stress–mediated checkpoint activation. Based on these considerations, docetaxel was selected as the chemotherapeutic agent for combination with verteporfin‐mediated SDT because of its potential synergistic interaction with SDT, established clinical efficacy, and manageable safety profile among taxane agents [14]. This combination integrates both cytotoxic and molecularly targeted mechanisms.

Cytotoxic chemotherapeutic agents, including taxanes, are small‐molecule drugs that undergo systemic distribution, frequently resulting in adverse events [21]. Liposomes are phospholipid bilayer‐based nanoparticles with amphiphilic properties that enable the encapsulation of both hydrophilic and hydrophobic compounds [22]. Nanoparticle‐based drug delivery systems, including liposomes and micelles, facilitate the preferential accumulation of encapsulated agents within tumor tissues through the enhanced permeability and retention effect [23]. This phenomenon is primarily attributed to the hyperpermeability of tumor‐associated vasculature and impaired lymphatic drainage within tumor tissues. Consequently, nanoparticle formulations are expected to enhance therapeutic efficacy while reducing systemic toxicity [23, 24].

Although synergistic antitumor effects of verteporfin‐mediated SDT combined with anticancer agents have been demonstrated in both in vitro and in vivo [14, 25], clinical evidence regarding this therapeutic approach remains limited. Therefore, in this study, we evaluated the combined effects of verteporfin‐mediated SDT and docetaxel in three patients with malignant tumors. Docetaxel and verteporfin were encapsulated in liposomes and administered as therapeutic agents. Encapsulation within liposomes of identical sizes is expected to facilitate comparable tumor accumulation.

## 2. Case Presentations

### 2.1. Study Design and Patients

We investigated the use of SDT in three patients with gastric cancer, ovarian metastases from gastric cancer, and lung cancer to evaluate whether SDT could be administered without inducing photosensitivity reactions or compromising their quality of life. The treatment timelines and clinical outcomes of the three patients are summarized in Table 1.

**Table 1:** Treatment timeline and clinical outcomes of the three patients.

Case	Patient	Timeline and treatment	CT assessment	Clinical response
1	47/F, gastric cancer with ovarian metastasis	2020: distal gastrectomy; 2022: ovarian metastasis; Day 0: docetaxel liposomes 20 mg + verteporfin liposomes 3.5 mg; Day 1: SDT (1 MHz, 2.0 W/cm^2^, 20 min); Day 8: verteporfin liposomes; Day 9: SDT	Baseline: 97.25 mm; Day 7: 97.82 mm; Day 42: 67.89 mm	PR; tumor became resectable and surgery was recommended.
2	49/F, recurrent breast cancer and pulmonary metastases	2019: neoadjuvant paclitaxel, AC, radiation therapy; 2022: pulmonary metastases, capecitabine + AC, followed by pembrolizumab/gemcitabine/carboplatin; Day 0: docetaxel liposomes 20 mg + verteporfin liposomes 3.5 mg; Day 1: SDT (3 MHz, 2.0 W/cm^2^, 20 min); Day 2: verteporfin liposomes; Day 6: Second treatment cycle.	Baseline: primary breast tumor 16.19 mm; Day 30: 10.56 mm	PR; treatment discontinued because of financial constraints.
3	92/M, gastric adenocarcinoma (cN2M0), unsuitable for surgery, radiotherapy, or chemotherapy	2023: diagnosed with gastric cancer. Day 0: docetaxel liposomes 20 mg + verteporfin liposomes 3.5 mg; Day 1: additional docetaxel liposomes 20 mg followed by SDT (1 MHz, 2.0 W/cm^2,^ 20 min). Day 6: second treatment cycle.	Day 43: improvement in posterior gastric wall thickening; response non‐evaluate because of differences in gastric distension between baseline and follow‐up CT.	Nonevaluable; no significant adverse events.

### 2.2. Docetaxel Liposomes and SDT Therapeutic Intervention Using Verteporfin Liposomes

Docetaxel and verteporfin liposomes were procured from StateArt Inc. (Tokyo, Japan). Verteporfin liposomes were diluted to 50 mL with 5% glucose solution (Hikari Seiyaku Ltd., Tokyo, Japan) and administered intravenously via the median cubital vein at a rate of 2 mL/min. Approximately 24 h after verteporfin liposome administration, SDT was performed using a therapeutic ultrasound system (PHYSIO SONO; Sakai Medical Co. Ltd., Tokyo, Japan). Ultrasound irradiation was applied to the pelvic region in the patient with ovarian metastasis from gastric cancer, the epigastric region in the patient with gastric cancer, and the bilateral upper lung fields in the patient with lung metastases from breast cancer. The nominal device settings were 1 MHz for the pelvic and epigastric regions and 3 MHz for the bilateral upper lung fields, with a displayed intensity of 2.0 W/cm^2^ for 20 min per treatment field. The acoustic parameters reported in this study represents the normal displayed settings of the ultrasound device. Because independent acoustic‐output calibration using a radiation‐force balance or a calibrated hydrophone was not performed for each treatment session, the actual in situ acoustic exposure could not be quantified retrospectively.

### 2.3. Photosensitivity Precautions and Monitoring

Because verteporfin is associated with transient cutaneous and ocular photosensitivity, all patients received standardized verbal instructions regarding light avoidance following each verteporfin liposome infusion. Patients were advised to avoid direct sunlight and intense indoor light exposure for 48 h after administration. When outdoor exposure was unavoidable, they were instructed to wear protective clothing and dark sunglasses. Patients were also informed that ultraviolet (UV) sunscreens alone do not prevent verteporfin‐related phototoxicity and that complete darkness was unnecessary, as exposure to normal indoor ambient light facilitates photobleaching of residual verteporfin. Throughout treatment and follow‐up, patients were monitored for photosensitivity‐related adverse events, including sunburn‐like erythema, edema, blistering, rash, ocular symptoms, infusion‐site reactions, and other light‐related symptoms.

### 2.4. Ethical Review and Informed Consent

This study was approved by the Ethics Review Committee of the Image Guided Therapy (IGT) Clinic on September 27, 2023 (Approval number: 27). Informed consent was obtained from all patients.

### 2.5. Clinical Findings

#### 2.5.1. Docetaxel Liposomes in Combination With SDT Using Verteporfin Liposomes for the Reduction of Krukenberg Tumors Secondary to Gastric Carcinoma

A 47‐year‐old woman with gastric cancer underwent distal gastrectomy involving approximately two‐thirds of the pyloric region at a community hospital in 2020. In 2022, ovarian metastases were detected, and the patient subsequently received 10 cycles of combination therapy with oxaliplatin and nivolumab at the same institution, resulting in an initial reduction in tumor size. However, tumor progression was subsequently observed, leading to treatment discontinuation. The patient then presented to our clinic seeking further treatment.

**Figure 1:**
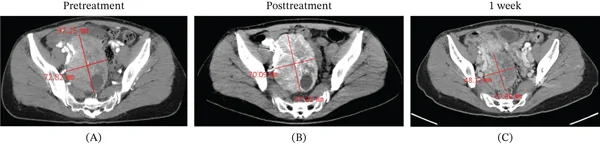
(A) Computed tomography (CT) image of the ovary in a patient with a Krukenberg tumor prior to treatment with docetaxel liposomes and sonodynamic therapy (SDT) using verteporfin liposomes. A large tumor is visible in the right ovary. (B) CT image obtained 1 day after the initial treatment shows no significant change. (C) CT image obtained 1 week after treatment demonstrates a marked reduction in tumor size.

Following confirmation of the lesion on computed tomography (CT) imaging, it measured 97.25 mm in the longest axial diameter. Docetaxel liposomes equivalent to 20 mg of docetaxel (14.5 mg/m^2^) and verteporfin liposomes equivalent to 3.5 mg of verteporfin (2.5 mg/m^2^) were administered intravenously. After 24 h, SDT was performed in the pelvic region using ultrasound at 1.0 MHz, 2.0 W/cm^2^ for 20 min. A CT scan obtained 1 week after treatment showed no further tumor growth, with the longest axial diameter measuring 97.82 mm. The following day, verteporfin liposomes (2.5 mg/m^2^) were administered intravenously, and SDT was repeated 24 h later. Subsequently, treatment was continued at regular intervals. A follow‐up CT scan performed 42 days after the initial administration demonstrated significant shrinkage of the ovarian tumor, with the longest axial diameter decreasing to 67.89 mm (Figures 1a–1c). The response was classified as partial response (PR). The tumor was considered resectable, and surgical resection was recommended as definitive treatment.

#### 2.5.2. Combined Docetaxel Liposomes and SDT Using Verteporfin Liposomes for Breast Cancer

A 49‐year‐old female patient was diagnosed with Stage II breast cancer of the right breast in 2019. She underwent chemotherapy with paclitaxel, followed by doxorubicin and cyclophosphamide (AC) therapy, and subsequently underwent radiation therapy. Following completion of treatment, no evidence of disease was observed. However, recurrent pulmonary metastases were detected in 2022. Despite treatment with capecitabine and AC therapy at a local hospital, the therapeutic response was limited. Two months later, the patient received pembrolizumab, gemcitabine, and carboplatin; however, the tumor continued to progress, and she was subsequently referred to our hospital.

**Figure 2:**
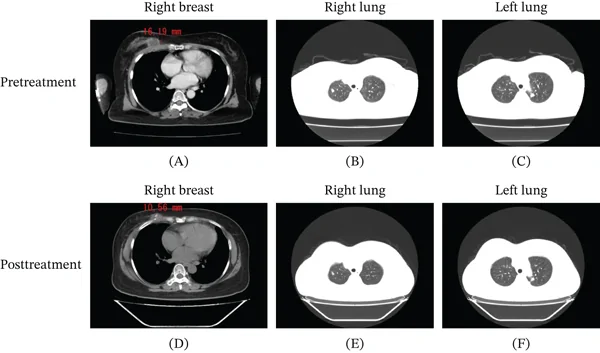
(A) CT image of the right breast tumor before treatment with docetaxel liposomes and SDT using verteporfin liposomes. (B, C) CT images of the right and left lungs, respectively, showing bilateral pulmonary metastases. (D−F) CT images of the right breast and bilateral lung fields obtained after treatment, demonstrating regression of the primary breast tumor and pulmonary metastatic legions.

The patient initially received intravenous administration of docetaxel liposomes equivalent to 20 mg of docetaxel (12.7 mg/m^2^) and verteporfin liposomes equivalent to 3.5 mg of verteporfin (2.2 mg/m^2^). At baseline, the primary breast tumor measured 16.19 mm in the longest axial diameter on CT. The following day, the same dose of docetaxel liposomes was administered. Subsequently, SDT was performed using a 3‐MHz probe applied to the right and left upper lung fields at an ultrasound intensity of 2.0 W/cm^2^ for 20 min. The same treatment protocol was repeated after 5 days. A CT scan obtained 1 month after treatment demonstrated a reduction in the size of both the primary breast tumor and the pulmonary metastatic lesions. The longest axial diameter of the primary breast tumor decreased from 16.19 mm to 10.56 mm (Figures 2a–2f), corresponding to a PR. Unfortunately, treatment was discontinued because of financial constraints.

#### 2.5.3. Combined Docetaxel Liposomes and SDT Using Verteporfin Liposomes for Gastric Cancer

A 92‐year‐old man was diagnosed with gastric cancer at our hospital in 2023 following a gallstone attack. Endoscopic biopsy of a lesion on the posterior wall of the stomach revealed high‐grade adenocarcinoma with regional lymph node involvement (N2) and no distant metastasis (M0). The serum carcinoembryonic antigen level was within the normal range (2.1 ng/mL). Given his advanced age, the patient was considered unsuitable for standard treatment modalities, including surgery, radiation therapy, and chemotherapy. He was then admitted to our hospital for further management.

Fluorodeoxyglucose positron emission tomography/CT demonstrated increased uptake corresponding to thickening of the posterior gastric wall. Docetaxel liposomes equivalent to 20 mg of docetaxel (12.6 mg/m^2^) and verteporfin liposomes equivalent to 3.5 mg of verteporfin (2.2 mg/m^2^) were administered intravenously. The following day, an additional 20 mg of docetaxel liposomes was administered intravenously. Subsequently, SDT was performed using a 1‐MHz probe applied to the epigastric region at an ultrasound intensity of 2.0 W/cm^2^ for 20 min. The patient was then transferred to a nearby hotel for convalescence. The same treatment protocol was repeated after 5 days.

**Figure 3:**
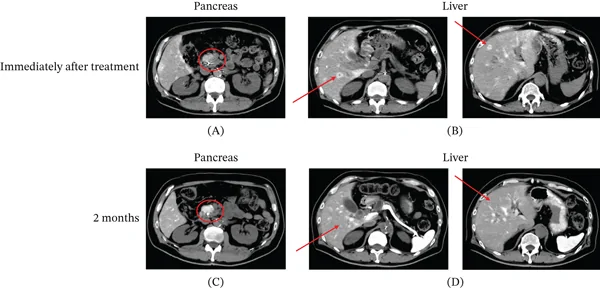
(A) Positron emission tomography and (B) CT images of the stomach obtained prior to treatment with docetaxel liposomes and SDT using verteporfin liposomes. Thickening of the posterior gastric wall is evident in both images. (C, D) CT images obtained from different planes approximately 1 month later, demonstrating a slight improvement in the posterior wall thickening. However, the treatment response was considered nonevaluate because of the discrepancy in gastric distension between the baseline and follow‐up CT examinations.

CT imaging performed 43 days after the initial administration showed an improvement in the thickening of the posterior gastric wall (Figures 3a−d). However, precise assessment of the degree of wall thickening was limited because of the discrepancy in gastric distension between the baseline and follow‐up imaging studies. Therefore, the treatment response of the gastric lesion was considered nonevaluate. No significant adverse events were observed during the interval between the pre‐ and post‐treatment CT examinations.

### 2.6. Photosensitivity‐Related Outcomes

No verteporfin‐related cutaneous or ocular phototoxicity was observed in any of the three patients during the treatment course of follow‐up period. Specifically, no patient developed sunburn‐like erythema, edema, blistering, rash, ocular symptoms, light‐related pain, or infusion‐site phototoxic reactions. In addition, no treatment delay, dose modification, or treatment discontinuation was required because of photosensitivity.

## 3. Discussion

The present study demonstrated the clinical application of docetaxel liposomes combined with verteporfin‐mediated SDT in patients with advanced malignancies, including gastric cancer with ovarian metastases, gastric cancer, and breast cancer with lung metastases. Although previous clinical studies have explored SDT in combination with anticancer agents, including the study by Wang et al. [26], those investigations evaluated different therapeutic regimens. To our knowledge, the present case series is the first clinical report describing the combination of verteporfin liposomes, liposomal docetaxel, and SDT.

Recent advances in SDT have increasingly focused on nanoparticle‐based drug delivery systems, multifunctional sonosensitizers, and combination strategies integrating chemotherapy and immunotherapy to enhance tumor selectivity and therapeutic efficacy while minimizing systemic toxicity. Furthermore, recent reviews have emphasized the growing interest in translating SDT into clinical oncology despite the limited availability of clinical evidence [27]. Therefore, the present cases series may provide clinically relevant evidence supporting the feasibility of verteporfin‐mediated SDT combined with liposomal docetaxel in patients with advanced, treatment‐refractory malignancies.

Patients with ovarian metastases from gastric cancer and lung metastases from breast cancer exhibited progressive disease despite prior treatment with anticancer agents and immune checkpoint inhibitors. However, tumor regression and/or growth suppression were observed following the present therapeutic intervention (Figures 1 and 3). One possible explanation for these therapeutic effects involves the inhibition of the interaction between YAP and TEAD transcription factors by verteporfin. The Hippo‐YAP signaling pathway regulates cellular proliferation and tumor progression. Under conditions of activated Hippo signaling, YAP1 is phosphorylated and retained in the cytoplasm through binding to 14‐3‐3 proteins, followed by degradation [28]. By contrast, unphosphorylated YAP translocates to the nucleus, where it binds to TEAD transcription factors and promotes the expression of genes associated with cell proliferation and tumor progression [29]. Previous studies have suggested that ATP‐binding cassette (ABC) transporters involved in drug efflux, including ABCB1 and ABCG2, are regulated by YAP−TEAD signaling [30–32]. In the present study, patients with breast cancer and lung metastases had previously developed resistance to paclitaxel, a taxane with a mechanism of action similar to that of docetaxel. Likewise, the patient with gastric cancer and ovarian metastases exhibited resistance to oxaliplatin‐based chemotherapy (Figure 1). Nevertheless, tumor growth suppression was observed following treatment in both cases. These findings suggest that the therapeutic effects observed in the present cases may be partially attributable to verteporfin‐mediated YAP1 inhibition, which may suppress ABC transporter expression and thereby reduce drugs efflux from tumor cells [30–32].

This therapeutic intervention may also modulate the immunosuppressive tumor microenvironment associated with YAP signaling. Previous studies have shown that YAP activation induces PD‐L1 expression and promotes the recruitment of immunosuppressive cells, including regulatory T cells, myeloid‐derived suppressor cells, and M2 macrophages, to the tumor microenvironment [31–35]. Consequently, the therapeutic responses observed in patients who had previously exhibited resistance to immune checkpoint inhibitors may be associated with enhanced antitumor immunity resulting from verteporfin‐mediated inhibition of YAP signaling.

The treatment administered to the older patient with gastric cancer demonstrated an acceptable safety profile and potential therapeutic benefit despite the patient′s advanced age. Because conventional intensive treatment was considered unsuitable, this therapeutic approach may represent a feasible treatment option for older patients who are ineligible for standard therapies.

Several limitations of the present case series should be acknowledged. First, because this was a retrospective case series including only three patients, the observed therapeutic responses cannot be distinguished definitively from the natural course of disease or the effects of prior treatments. Second, the ultrasound exposure parameters were reported as the nominal displayed settings of the therapeutic ultrasound device. Independent acoustic‐output verification using a radiation‐force balance or a calibrated hydrophone was not performed for each treatment session; therefore, the actual in situ acoustic exposure could not be quantified retrospectively. Future prospective studies should incorporate standardized acoustic dosimetry, including measurements of acoustic power, effective radiating area, spatial‐average temporal‐average intensity, duty cycle, beam profile, coupling conditions, and temperature monitoring. Third, no mechanistic analyses, including evaluation of YAP1 activity, ABC transporter expression, clinical immunophenotyping, or ICD‐related biomarkers (e.g., calreticulin exposure, HMGB1 release, and ATP release), were performed. Therefore, the proposed mechanisms should be regarded as hypotheses requiring validation in future translational studies.

## 4. Conclusions

Although the precise mechanisms underlying the observed therapeutic effects remain to be fully elucidated, verteporfin‐mediated inhibition of YAP signaling and modulation of the tumor immune microenvironment may contribute to the antitumor response. Further clinical studies involving larger patient populations, together with translational analyses using human tumor specimens, are warranted to clarify both the therapeutic efficacy and the underlying mechanisms of this treatment strategy.

**Nomenclature**
ABC: ATP‐binding cassette
CT: computed tomography
ICD: immunogenic cell death
PDT: photodynamic therapy
PD‐L1: programmed death ligand 1
SDT: sonodynamic therapy
YAP: yes‐associated protein
TEAD: transcriptional enhanced associate domain

## Author Contributions

Conceptualization, Y. K. and S. K.; methodology, Y. K.; software, S. K.; validation, Y. K., S. K., Y. H., S. F., and M. T.; formal analysis, Y. K. and S. K.; investigation, Y. K. and S. K.; resources, Y. K.; data curation, S. K.; writing—original draft preparation, S. K.; writing—review and editing, R. T., O. I., T. Y., and K. H.; visualization, M. G.; supervision, O. I., T. Y., and K. H.; project administration, Y. K. Y. K. and S. K contributed equally.

## Funding

No funding was received for this manuscript

## Disclosure

All authors have read and agreed to the published version of the manuscript.

## Ethics Statement

This study was conducted in accordance with the Declaration of Helsinki and the Ethical Guidelines for Medical Research Involving Human Subjects established by the Japanese Ministry of Health, Labor and Welfare, and approved by the Ethics Review Committee at the IGT Clinic (approval number: 27 and date of approval: September 27, 2023).

## Consent

Written informed consent was obtained from all patients for the publication of this paper.

## Conflicts of Interest

The authors declare no conflicts of interest.

## Data Availability

The data that support the findings of this study are available from the corresponding author upon reasonable request.
